# The Efficacy of Mannitol in Attenuating Postreperfusion Syndrome in Orthotopic Liver Transplantation: A Retrospective Cohort Study †

**DOI:** 10.3390/jcm14061897

**Published:** 2025-03-11

**Authors:** Samuel DeMaria, Jr., Emily M. Bachner, Victoria Mroz, Sophia Gamboa, Yuxia Ouyang, Natalia N. Egorova, Natalie K. Smith, Ryan Wang

**Affiliations:** 1 Department of Anesthesiology, Perioperative and Pain Medicine, The Icahn School of Medicine at Mount Sinai Hospital, New York, NY 10029, USA; samuel.demaria@mssm.edu (S.D.J.);; 2 Icahn School of Medicine at Mount Sinai, New York, NY 10029, USA; 3 Department of Population Health Science and Policy, The Icahn School of Medicine at Mount Sinai Hospital, New York, NY 10029, USA

**Keywords:** anesthesiology, liver transplant, reperfusion syndrome, perioperative care, acute renal failure

## Abstract

**Introduction:** Postreperfusion syndrome (PRS) is associated with complications following liver transplantation (LT). Mannitol may play a role in attenuating PRS as a free radical scavenger. This study aimed to evaluate the association between intraoperative mannitol administration and the incidence of PRS and postoperative acute kidney injury (AKI) in LT. **Methods:** A retrospective analysis of adult liver-only transplantation between August 2019 and January 2023 at the Mount Sinai Hospital was performed. Patients in the mannitol group received 25G of the drug intravenously prior to reperfusion. Any recipients with pre-existing renal diagnoses were excluded. Demographic, laboratory, intraoperative, and hospital course data were extracted from an institutional data warehouse. Multivariable logistic regressions were used to evaluate the association between mannitol administration and PRS, AKI, early allograft dysfunction, and postoperative cardiac complications. Negative binomial regression was used to evaluate the association with postoperative length of stay (LOS) and ICU LOS. **Results:** 495 LT cases were included. A total of 81 patients received mannitol before graft reperfusion, while 414 patients did not. The incidence of PRS in patients who received mannitol was 13% and 17% for those who did not receive mannitol (*p* = 0.53). Additionally, 79% of patients who received mannitol experienced AKI at 7 days, compared to 73% in those who did not receive mannitol (*p* = 0.48). In the multivariable regression models, mannitol administration was not associated with decreased incidence of PRS or postoperative AKI. It was, however, associated with increased postoperative cardiac complications (risk-adjusted odds ratio 2.70, 95% confidence interval 1.15–6.14, *p* = 0.02). **Conclusions:** Mannitol administration during LT was not an effective therapy for reducing PRS or postoperative AKI.

## 1. Introduction

In liver transplantation (LT), postreperfusion syndrome (PRS) is widely recognized as an early sign of severe ischemia–reperfusion injury in the graft. PRS is characterized by a constellation of hemodynamic changes such as hypotension, arrhythmias, and systemic inflammation after the restoration of blood flow to a transplant graft. It has been estimated that 25–60% of patients who undergo LT will experience PRS (generally defined as a decrease in the mean arterial pressure (MAP) by 30% or more within 5 min following reperfusion and lasting approximately 1 min or longer) [1,2,3,4]. PRS has also been found to be associated with postoperative acute kidney injury (AKI), early graft failure, and overall worsening of clinical outcomes [5,6,7]. Key risk factors for PRS are numerous and include recipient advanced age, high model for end-stage liver disease score (MELD), prolonged ischemia times, and pre-existing cardiac dysfunction. Older donor age, donation after circulatory death (DCD) status, and steatosis can also influence PRS [3,4].

Oxygen free radicals have been recognized as one of the potential contributors to PRS [8]; therefore, identifying a method to reduce local reactive oxygen species may prove beneficial in the prevention of PRS in LT [8]. Mannitol is an osmotic diuretic with a proposed antioxidative capacity in other tissues, and the use of mannitol during kidney transplantation has been found to reduce the risk of both AKI and delayed graft function [9,10,11]. It is possible mannitol could have beneficial use in preventing PRS via free radical scavenging and/or its effects on increasing intravascular free water volume. However, its effect on PRS during LT has yet to be fully elucidated. There have been small studies evaluating mannitol’s ability to decrease the risk of PRS in LT, but with variable results [12,13]. This study seeks to investigate the relationship between intraoperative mannitol administration and the occurrence of PRS in the context of liver transplantation.

## 2. Materials and Methods

This retrospective cohort study was approved by the Program for the Protection of Human Subjects at the Mount Sinai Hospital with a waiver of consent (no. 19-02605) and was performed in accordance with the Declaration of Helsinki. Adult patients who underwent living donor or deceased donor liver transplant at our institution from August 2019 (the start of data collection in this data warehouse) to January 2023 were included. Patients were identified by querying the departmental data warehouse for patients who underwent liver transplantation based on current procedural terminology (CPT) coding (CPT = 47,135). Patients less than 18 years old, those with pre-existing renal diagnoses, or patients who underwent simultaneous transplantation of other organs were excluded. Patients were then divided into two cohorts: those who received mannitol intraoperatively and those who did not. All patients underwent a standard anesthetic approach at our institution, including propofol for induction of anesthesia, isoflurane maintenance, standard ASA monitoring plus pulmonary artery catheterization, transesophageal echocardiography, and a radial arterial line. One 9Fr central line and a peripheral 7Fr rapid infusion catheter were placed as well. All patients underwent a total caval replacement surgical approach and received 500 mg methylprednisolone after the hepatic artery was re-anastomosed. Postoperatively, patients were brought to a dedicated transplant ICU intubated and mechanically ventilated. Immunosuppression in the ICU included continued methylprednisolone as well as tacrolimus and mycophenolate mofetil. Mannitol administration was at the discretion of the attending anesthesiologist. In each case where it was administered, 25G of mannitol was given intravenously over approximately 30 min, starting when the initial surgical clamping of the upper vena cava occurred (Pfanstiehl, Inc., Lake Forest, IL, USA).

Demographic data were extracted from the departmental data warehouse. Comorbidity data, including history of arrhythmias, congestive heart failure, or coronary artery disease, were extracted from patient problem lists present on admission using International Classification of Diseases-10 (ICD-10) codes. Similarly, postoperative cardiac and renal complications were defined as having new diagnoses added to their problem list within 90 days of liver transplant within the following range of ICD-10 codes: I20-79, N10-19, N99. Etiologies and complications of liver disease were extracted. Missing data were filled in whenever possible by manual chart review. Postoperative length of stay (LOS) was the total number of days present in the hospital after transplant. Postoperative intensive care unit (ICU) LOS was defined as the number of days the patient was admitted to the ICU postoperatively, including potential readmissions to the ICU during the hospital stay. Preoperative baseline laboratory values were defined as the latest lab values collected prior to arriving at the operating room for liver transplantation. Using these preoperative baseline lab values, the MELD-Na score was calculated as described in the literature [14]. PRS was defined as a drop in mean arterial pressure (MAP) by at least 30% within 5 min of organ reperfusion and lasting for at least 1 min [2]. The standard surgical and anesthetic management of liver transplantation at our institution has been previously described [15]. Over the study period, mannitol was given by two out of eight dedicated liver transplant anesthesia attendings as part of our overall efforts to improve hemodynamic stability at reperfusion, which included pre-emptive and aggressive treatment to normalize serum potassium and calcium prior to reperfusion as well as use of vasopressors to optimize blood pressure and heart rate before reperfusion; all interventions were performed at the discretion of the attending anesthesiologist. Mannitol was avoided in patients who would not benefit from intravascular volume expansion (e.g., portopulmonary syndrome, signs of fluid overload, reduced ventricular function).

The primary outcome was the incidence of PRS. Secondary outcomes included incidence of early allograft dysfunction (EAD) and incidence of AKI. EAD was defined as (1) total bilirubin ≥ 10 mg/dL on post-op day 7, (2) INR ≥ 1.6 on post-op day 7, and (3) ALT or AST > 2000 IU/mL within the first 7 days post liver transplant [16]. AKI was defined as either an increase in serum creatinine by ≥0.3 mg/dL or an increase in serum creatinine to ≥1.5 times baseline [17]. This was reported at 48 h and at seven days postoperatively. Additional secondary outcomes included postoperative cardiac complications, postoperative hospital and ICU LOS, and in-hospital mortality.

## 3. Statistical Analyses

All variables were summarized using the appropriate descriptive statistics. All continuous variables were visually inspected. Continuous variables were presented as median [interquartile range], and categorical variables were presented as count (percentage).

The *t*-test and Kruskal–Wallis test were used for numerical variables, and the chi-square or Fisher’s exact test was used for categorical variables, as appropriate, to assess differences between the two study cohorts: those who did and those who did not receive mannitol intraoperatively. *p*-values < 0.05 were considered significant. Logistic regression models for binary outcomes (i.e., PRS, EAD, AKI within 48 h, and AKI at 7 days) and negative binomial regression models for postoperative and ICU LOS were used to assess the associations of the outcomes with mannitol administration, controlling for the recipient’s sex, race, BMI, MELD-Na, baseline lab values (i.e., hematocrit, potassium, creatinine), donor age, donor BMI, donor macrosteatosis (i.e., mild, moderate), cold ischemia time (CIT), warm ischemia time (WIT), machine perfusion, donor type (i.e., donation after circulatory death (DCD), donation after brain death (DBD), or living donor), and re-operation. The covariates were determined based on their clinical significance. Statistical analyses were performed using R v3.6.1 in RStudio v1.2.5001 (RStudio, Boston, MA, USA).

## 4. Guidelines

The authors followed the STROBE guidelines for conducting this study [18].

## 5. Results

**Figure 1:**
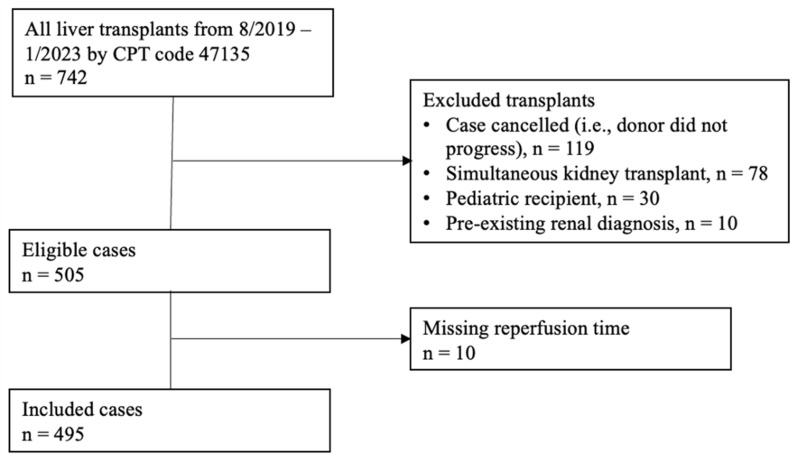
Patient selection diagram.

Of the 739 liver transplant cases identified from August 2019 to January 2023 at our single-center institution, there were 495 patients that met the inclusion criteria (Figure 1). A total of 81 (16.4%) cases received intraoperative mannitol prior to reperfusion, and 414 (83.6%) did not. The baseline characteristics of the included patients are summarized in Table 1. Patients were predominantly male (62%) with a median age of 57 [47–64] years. The median MELD-Na score was 26 [14–33]. The median baseline creatinine was 0.96 [0.71–1.50] mg/dL. Notably, there was a difference in the baseline creatinine levels between patients who received mannitol compared to patients who did not (0.82 vs. 1.01 mg/dL, *p* = 0.02). A minority of patients had a history of acute coronary syndrome (4.6%), congestive heart failure (1.2%), or arrhythmia (4.4%) prior to liver transplant. The median donor age was 41 [31–51] years old. There were 52 (13%) DCD grafts in the no-mannitol group, while there were 22 (27%) DCD grafts in the mannitol group, *p* = 0.003.

The incidence of PRS in patients who received mannitol was 11 (13.6%), compared to 71 (17.1%) in the group that did not (*p* = 0.53) (Table 2). Among the patients who were administered mannitol, 266 (64.3%) developed AKI within 48 h, compared to 60 (74.1%) in those who did not receive mannitol (*p* = 0.18). Additionally, 64 (79.0%) of patients who received mannitol experienced AKI at 7 days compared to 302 (72.9%) in those who did not receive mannitol (*p* = 0.48). Patients who received intraoperative mannitol had a significantly higher incidence of postoperative cardiac complications compared to those who did not (18 (22%) vs. 52 (12.6%), *p* = 0.04) and a significantly higher incidence of EAD (13 (16%) vs. 30 (7%), *p* = 0.02). There were no significant differences between the two groups for postoperative hospital LOS (11.5 [6–25.3] vs. 9 [5–18.3] days, *p* = 0.09) or ICU LOS (6 [4–14] vs. 5 [3–9] days, *p* = 0.05) on univariate [4–14] analysis.

In multivariable regression models, patients who received mannitol had an increased odds of postoperative cardiac complications (risk-adjusted OR 2.70, 95% CI (confidence interval) 1.15–6.14, risk adjusted *p* = 0.02) compared to the control group by logistic regression (Table 3). Mannitol administration was associated with longer postoperative hospital [4–14 LOS (20.9 vs. 17.4 days, OR 1.51, 95% CI 1.20–1.91, risk adjusted *p* < 0.001) by negative binomial regression (Table 3). There was no association between mannitol administration and PRS, EAD, AKI at 48 h, AKI at 7 days, postoperative ICU LOS, or in-hospital mortality. When subjected to propensity matching, mannitol administration was not associated with PRS, EAD, AKI, postoperative ICU LOS, hospital length of stay, cardiac complications, or in-hospital mortality (all *p* > 0.2).

## 6. Discussion

In this single-center retrospective cohort study, we observed no significant association between intraoperative mannitol administration and the development of PRS or postoperative AKI in liver transplant recipients. However, there were found to be elevated rates of postoperative cardiac complications as well as prolonged hospitalization and ICU stays in the mannitol group. On propensity matching, these differences did not persist.

There was no significant difference found in PRS incidence between the two cohorts of the study. Emara et al. also found comparable rates of PRS among living donor liver transplant recipients who received and did not receive mannitol [19]. This study extends those findings to deceased donor liver transplantation. In contrast, Sahmeddini et al. examined the change in hemodynamics of patients who received mannitol during the anhepatic phase of liver transplantation and found that these patients did not experience the same decrease in MAP as the control group [12]. However, this study did not report PRS rates, and the mean decrease in MAP observed in the study was less than the 30% decrease that is commonly used to define PRS. Additionally, Sahmeddini et al. excluded patients who received a blood transfusion prior to reperfusion, despite the literature suggesting higher transfusion requirements to be a risk factor for PRS [3,5].

This study also found no association between mannitol administration and the development of AKI after liver transplantation. These findings are consistent with those reported by Emara et al. and Whitta et al. [19,20]. A meta-analysis by Yang et al. found that while mannitol administration for kidney transplant recipients was associated with decreased delayed kidney graft function and AKI rates, similar renal protective effects were not seen in non-kidney transplant recipients, which might help further explain why mannitol was not found to be protective against post-LT AKI in this study [21].

One notable association was that between mannitol administration and DCD grafts. To account for possible confounding, DCD graft use was included in the multivariable regression models to adjust for its impact on the outcomes. Additionally, Chadha et al. did not find DCD graft use to be associated with PRS or postoperative need for renal replacement therapy [22], making it less likely that the association of mannitol with DCD grafts impacted the PRS or postoperative AKI rates in this study.

Another noteworthy finding of this study was the increased risk of postoperative cardiac complications associated with mannitol administration, even after accounting for the impact of DCD grafts, MELD-Na score, and recipient age in the regression model. Prior studies have identified higher MELD scores, intraoperative transfusion requirements, and recipient age as risk factors for post-LT cardiac complications [23,24]. It is possible for mannitol administration to cause elevated intravascular volume and heart failure if not carefully monitored. In a study of traumatic brain injury patients receiving mannitol, Dabrowski et al. found increased plasma osmolality was associated with QT prolongation and an increased incidence of atrial fibrillation [25]. Due to the retrospective nature of this study, the results cannot distinguish between mannitol administration increasing the risk of postoperative cardiac dysfunction or if mannitol is only associated with other mediating factors. Regardless, the impact of mannitol on post-LT cardiac outcomes should be further studied as cardiac complications significantly impact the post-transplant survival of liver transplant recipients [26]. These complications did not show an association after propensity score matching, but caution in interpreting these results is advised given the small size of the experimental group as well as the pre-existing presence of well-matched demographics prior to such matching.

An increase in postoperative hospital LOS was also associated with mannitol administration in this study. However, given the main limitations of this study (i.e., its retrospective nature and the non-protocolized administration of mannitol) and the many variables that impact LOS after LT [27], this finding requires additional study. This study’s generalizability is limited due to its single-center design, as healthcare practices and patient characteristics can vary across institutions. However, the findings raise questions regarding the use of 25% mannitol during LT. Its administration was not associated with PRS reduction or renal protection, but it was associated with postoperative cardiac complications. Further prospective investigations are warranted, and they should factor possible postoperative complications into their study designs.

## Figures and Tables

**Table 1:** Baseline characteristics of patients by mannitol administration.

Variable	No Mannitol (*n* = 414)	Mannitol (*n* = 81)	*p*-Value	All Patients (*n* = 495)
Age (years)	57.0 [47.0–64.0]	57.0 [48.0–65.0]	0.68	57.0 [47.0–64.0]
Sex			0.09	
Male	264 (63.8)	43 (53.1)		307 (62.0)
Female	150 (36.2)	38 (46.9)		188 (38.0)
Race			0.54	
White	196 (47.3)	32 (39.5)		228 (46.1)
Asian	39 (9.4)	7 (8.6)		46 (9.3)
Black	39 (9.4)	10 (12.3)		49 (9.9)
Other	140 (33.8)	32 (39.5)		172 (34.7)
BMI (kg/m^2^)	27.7 [24.8–31.8]	26.4 [23.6–31.5]	0.19	27.5 [24.7–31.8]
ASA Classification			0.45	
2	1 (0.2)	1 (1.2)		2 (0.4)
3	5 (1.2)	1 (1.2)		6 (1.2)
4	391 (94.4)	75 (92.6)		466 (94.1)
5	17 (4.1)	4 (4.9)		21 (4.2)
Baseline lab values				
Hematocrit (%)	30.6 [26.1–35.8]	31.5 [25.6–36.9]	0.63	30.7 [26.1–36.0]
Potassium (mEq/L)	4.1 [3.7–4.4]	3.9 [3.6–4.3]	0.21	4.0 [3.7–4.4]
Sodium (mEq/L)	137 [133–137]	137 [133–137]	0.67	137 [133–137]
Creatinine (mg/dL)	1.01 [0.72–1.55]	0.82 [0.65–1.33]	**0.02**	0.96 [0.71–1.50]
Bilirubin (mg/dL)	5.8 [1.9–19.4]	6.1 [2.1–21.2]	0.98	5.9 [1.9–19.7]
INR	1.9 [1.4–2.8]	1.9 [1.4–2.6]	0.88	1.9 [1.4–2.8]
MELD-Na	26 [14–34]	26 [14–31]	0.51	26 [14–33]
Preoperative Cardiac Comorbidities				
CAD	19 (4.6)	4 (4.9)	0.78	23 (4.6)
CHF	5 (1.2)	1 (1.2)	1.00	6 (1.2)
Arrhythmia	17 (4.1)	5 (6.2)	0.38	22 (4.4)
Diabetes	57 (13.7)	10 (12.3)	0.59	67 (13.5)
Hypertension	122 (29.5)	25 (31.0)	0.27	147 (29.7)
Prior Liver Transplant	22 (5.3)	1 (1.2)	0.15	23 (4.6)
Donor Variables				
BMI (kg/m^2^)	26.4 [23.4–31.1]	26.3 [23.2–30.5]	0.76	26.4 [23.3–30.9]
Age (years)	42.5 [31.0–51.8]	39.0 [32.5–48.0]	0.24	41.0 [31.0–51.0]
CIT (min)	330 [270–390]	321 [273–360]	0.51	326 [270–388]
WIT (min)	31.0 [26.0–37.0]	30.0 [25.0–34.0]	0.43	31.0 [26.0–36.5]
Normothermic Machine Perfusion Use	28 (6.8)	0 (0)	**0.01**	28 (5.7)
Donor Steatosis			0.17	
None	368 (88.9)	68 (84.0)		436 (88.1)
Mild	39 (9.4)	10 (12.3)		49 (9.9)
Moderate	7 (1.7)	3 (3.7)		10 (2.0)
Donor Type			**0.003**	
DBD	331 (80.0)	55 (67.9)		386 (78.0)
DCD	52 (12.6)	22 (27.2)		74 (14.9)
LD	31 (7.5)	4 (4.9)		35 (7.1)

Data are as provided number (percentage) or median [interquartile range]. *p* < 0.05 are bolded. Abbreviations: BMI, body mass index; CAD, coronary artery disease; CHF, congestive heart failure; CIT, cold ischemic time; DBD, donation after brain death; DCD, donation after circulatory death (all donors were controlled DCD, occurring in the donor site operating room); LD, living donor; WIT, warm ischemic time. Donor steatosis was determined by pre-procurement histology.

**Table 2:** Outcome variables by mannitol administration.

	No Mannitol (*n* = 414)	Mannitol (*n* = 81)	*p*-Value	Total (*n* = 495)
PRS	71 (17.1)	11 (13.6)	0.53	82 (16.6)
AKI at 48 h	266 (64.3)	60 (74.1)	0.18	326 (65.9)
AKI at 7 days	302 (72.9)	64 (79.0)	0.48	366 (73.9)
Postoperative cardiac complications	52 (12.6)	18 (22.2)	**0.04**	70 (14.1)
EAD	30 (7.2)	13 (16.0)	**0.02**	43 (8.7)
Postoperative LOS (days)	9 [5–18]	12 [6–25]	0.09	9 [5–19]
Postoperative ICU LOS (days)	5 [3–10]	6 [4–10]	0.15	5 [3–10]
In-hospital mortality	23 (5.6)	1 (1.2)	0.10	24 (4.8%)

Data are as provided number (percentage) or median [interquartile range]. *p* < 0.05 are bolded. Abbreviations: AKI, acute kidney injury; EAD, early allograft dysfunction; ICU, intensive care unit; LOS, length of stay; PRS, postreperfusion syndrome.

**Table 3:** The association between mannitol administration and outcomes based on the results of multivariable regression analysis.

Outcome	Odds Ratio	Confidence Interval	*p*-Value
PRS	0.67	0.25–1.57	0.38
AKI at 48 h	1.94	0.90–4.44	0.10
AKI at 7 days	1.63	0.72–4.06	0.26
Postoperative Cardiac Complication	2.70	1.15–6.14	**0.02**
EAD	2.35	0.82–6.32	0.10
Postoperative LOS *	1.61	1.29–2.03	**<0.001**
Postoperative ICU LOS *	1.23	0.88–1.74	0.22
In-Hospital Mortality	0	0.00–Inf	0.99

Logistic regression was used for binary outcomes. * Negative binomial regression was used for continuous variables LOS and ICU LOS. *p* < 0.05 are bolded. Abbreviations: AKI, acute kidney injury; EAD, early allograft dysfunction; ICU, intensive care unit; LOS, length of stay; PRS, postreperfusion syndrome.

## Data Availability

The original contributions presented in this study are included in the article. Further inquiries can be directed to the corresponding author.

## Abbreviations

AKI, acute kidney injury; BMI, body mass index; CIT, cold ischemia time; CPT, current procedural terminology; DBD, donation after brain death; DCD, donation after circulatory death; ICD, International Classification of Diseases; ICU, intensive care unit; LOS, length of stay; LD, living donor; LT, liver transplantation; MAP, mean arterial pressure; MELD-Na, model for end-stage liver disease-sodium; PRS, postreperfusion syndrome; WIT, warm ischemia time.

## References

[1] Lee J., Yoo Y.J., Lee J.M., Park Y.J., Ryu H.G. (2016). Sevoflurane versus desflurane on the incidence of postreperfusion syndrome during living donor liver transplantation: A randomized controlled trial. Transplantation.

[2] Aggarwal S., Kang Y., Freeman J., Fortunato F.L., Pinsky M. (1993). Postreperfusion Syndrome: Hypotension After Reperfusion of the Transplanted Liver. J. Crit. Care.

[3] Bukowicka B., Akar R.A., Olszewska A., Smoter P., Krawczyk M. (2011). The occurrence of postreperfusion syndrome in orthotopic liver transplantation and its significance in terms of complications and short-term survival. Ann. Transplant..

[4] Sahmeddini M.A., Tehran S.G., Khosravi M.B., Eghbal M.H., Asmarian N., Khalili F., Vatankhah P., Izadi S. (2022). Risk factors of the post-reperfusion syndrome during orthotopic liver transplantation: A clinical observational study. BMC Anesthesiol..

[5] Hilmi I., Horton C.N., Planinsic R.M., Sakai T., Nicolau-Raducu R., Damian D., Gligor S., Marcos A. (2008). The Impact of Postreperfusion Syndrome on Short-Term Patient and Liver Allograft Outcome in Patients Undergoing Orthotopic Liver Transplantation. Liver Transplant..

[6] Varotti G., Grazi G.L., Vetrone G., Ercolani G., Cescon M., Del Gaudio M., Ravaioli M., Cavallari A., Pinna A. (2005). Causes of early acute graft failure after liver transplantation: Analysis of a 17-year single-centre experience. Clin. Transplant..

[7] Barri Y.M., Sanchez E.Q., Jennings L.W., Melton L.B., Hays S., Levy M.F., Klintmalm G.B. (2009). Acute Kidney Injury Following Liver Transplantation: Definition and Outcome Yousri. Liver Transplant..

[8] Li C., Jackson R.M. (2002). Reactive species mechanisms of cellular hypoxia-reoxygenation injury. Am. J. Physiol..

[9] Larsen M., Webb G., Kennington S., Kelleher N., Sheppard J., Kuo J., Unsworth-White J. (2002). Mannitol in cardioplegia as an oxygen free radical scavenger measured by malondialdehyde. Perfusion.

[10] Reiterer C., Hu K., Sljivic S., Falkner von Sonnenburg M., Fleischmann E., Kabon B. (2021). The effect of mannitol on oxidation-reduction potential in patients undergoing deceased donor renal transplantation—A randomized controlled trial. Acta Anaesthesiol. Scand..

[11] van de Laar S.C., Schouten G.N., IJzermans J.N.M., Minnee R.C. (2021). Effect of Mannitol on Kidney Function After Kidney Transplantation: A Systematic Review and Meta-Analysis. Transplant. Proc..

[12] Sahmeddini M., Zahiri S., Khosravi M., Ghaffaripour S., Eghbal M., Shokrizadeh S. (2014). Effect of mannitol on postreperfusion cardiac output and central venous oxygen saturation during orthotopic liver transplant: A double-blind randomized clinical trial. Prog. Transplant..

[13] Weinbroum A.A., Shapira I., Abraham R.B., Szold A. (2002). Mannitol dose-dependently attenuates lung reperfusion injury following liver ischemia reperfusion: A dose-response study in an isolated perfused double-organ model. Lung.

[14] Kim W.R., Biggins S.W., Kremers W.K., Wiesner R.H., Kamath P.S., Benson J.T., Edwards E., Therneau T.M. (2015). Hyponatremia and Mortality among Patients on the Liver-Transplant Waiting List. N. Engl. J. Med..

[15] Bekki Y., Myers B., Wang R., Smith N., Zerillo J., Rocha C., Tabrizian P., Moon J., Arvelakis A., Facciuto M.E. (2022). Postreperfusion syndrome in liver transplantation: Outcomes, predictors, and application for recipient selection. Clin. Transplant..

[16] Olthoff K.M., Kulik L., Samstein B., Kaminski M., Abecassis M., Emond J., Shaked A., Christie J.D. (2010). Validation of a Current Definition of Early Allograft Dysfunction in Liver Transplant Recipients and Analysis of Risk Factors. Liver Transplant..

[17] Palevsky P.M., Liu K.D., Brophy P.D., Chawla L.S., Parikh C.R., Thakar C.V., Tolwani A.J., Waikar S.S., Weisbord S.D. (2013). KDOQI US commentary on the 2012 KDIGO clinical practice guideline for acute kidney injury. Am. J. Kidney Dis..

[18] von Elm E., Altman D.G., Egger M., Pocock S.J., Gøtzsche P.C., Vandenbroucke J.P. (2008). The Strengthening the Reporting of Observational Studies in Epidemiology (STROBE) statement: Guidelines for reporting observational studies. J. Clin. Epidemiol..

[19] Emara M.M., Diab D.G., Yassen A.M., Abo-Zeid M.A. (2022). Mannitol for prevention of acute kidney injury after liver transplantation: A randomized controlled trial. BMC Anesthesiol..

[20] Whitta R.K.S., Marshall C., Bates S., Appleby J. (2001). Intraoperative Mannitol Does Not Prevent Renal Failure in Orthotopic Liver Transplantation. Crit. Care Resusc..

[21] Yang B., Xu J., Xu F., Zou Z., Ye C., Mei C., Mao Z. (2014). Intravascular administration of mannitol for acute kidney injury prevention: A systematic review and meta-analysis. PLoS ONE.

[22] Chadha R.M., Croome K.P., Aniskevich S., Pai S., Nguyen J., Burns J., Perry D., Taner C.B. (2019). Intraoperative Events in Liver Transplantation Using Donation After Circulatory Death Donors. Liver Transplant..

[23] Sharma S., Sonny A., Dalia A.A., Karamchandani K. (2020). Acute heart failure after liver transplantation: A narrative review. Clin. Transplant..

[24] Yataco M.L., Difato T., Bargehr J., Rosser B.G., Patel T., Trejo-Gutierrez J.F., Pungpapong S., Taner C.B., Aranda-Michel J. (2014). Reversible non-ischaemic cardiomyopathy and left ventricular dysfunction after liver transplantation: A single-centre experience. Liver Int..

[25] Dabrowski W., Siwicka-Gieroba D., Robba C., Badenes R., Bialy M., Iwaniuk P., Schlegel T.T., Jaroszynski A. (2020). Plasma hyperosmolality prolongs qtc interval and increases risk for atrial fibrillation in traumatic brain injury patients. J. Clin. Med..

[26] James T.W., Shay J.E.S., Furfaro D., Ozseker B., Russell S.D., Pustavoitau A., Rizkalla N., Saberi B., Philosophe B., Cameron A.M. (2019). Cardiac events within the 30-day postoperative period is associated with increased 1-year mortality among deceased-donor liver transplant recipients. Exp. Clin. Transplant..

[27] Pita A., Nguyen B., Rios D., Maalouf N., Lo M., Genyk Y., Sher L., Cobb J.P. (2019). Variability in intensive care unit length of stay after liver transplant: Determinants and potential opportunities for improvement. J. Crit. Care.

